# Project Resenha da Prevenção and drug use: adolescents’ perceptions from a socio ecological perspective

**DOI:** 10.1590/0034-7167-2025-0342

**Published:** 2026-11-06

**Authors:** Brenda Pinheiro Evangelista, Thais Rodrigues de Albuquerque, Kirley Kethellen Batista Mesquita, Mariana Sales Bastos, Priscila de Souza Aquino, Lorena Pinheiro Barbosa, Patrícia Neyva da Costa Pinheiro, Fabiane do Amaral Gubert

**Affiliations:** I Universidade Federal do Ceará. Fortaleza, Ceará, Brazil

**Keywords:** Health Promotion, Adolescent, Child, Sports and Recreational Facilities, Primary Prevention., Promoción de la Salud, Adolescente, Niño, Instalaciones Deportivas y Recreativas, Prevención Primaria.

## Abstract

**Objectives:**

to analyze adolescents’ perceptions of their participation in the *Resenha da Prevenção* Project and their views on alcohol and other drug use, from the perspective of the Socio Ecological Model.

**Methods:**

a qualitative study conducted with 97 adolescents aged 10 to 19 years, enrolled in the *Resenha da Prevenção* project in four municipalities in the state of Ceará, Brazil, between May and June 2025. Data were collected through focus group techniques, subjected to reflexive thematic analysis, and interpreted using the Socio Ecological Model.

**Results:**

two central themes emerged: Perceptions of alcohol and other drug use, and Perceptions of outcomes and challenges related to participation in the *Resenha da Prevenção* Project. The interpersonal level of influence was predominant.

**Final Considerations:**

in the adolescents’ view, the *Resenha da Prevenção* project fosters personal and social development by providing a safe space for learning and reflection.

## RESUMEN

**Objetivos:**

analizar las percepciones de los adolescentes sobre su participación en el Proyecto *Resenha da Prevenção* y sobre el consumo de alcohol y otras drogas, desde la perspectiva del Modelo Socioecológico.

**Métodos:**

estudio cualitativo realizado con 97 adolescentes de 10 a 19 años, inscritos en el Proyecto *Resenha da Prevenção* en cuatro municipios del estado de Ceará, Brasil, entre mayo y junio de 2025. Los datos, recolectados mediante la técnica de grupo focal, fueron sometidos a análisis temático reflexivo y examinados a partir del Modelo Socioecológico.

**Resultados:**

surgieron dos temas centrales: Percepción sobre el consumo de alcohol y otras drogas, y Percepciones sobre los resultados y desafíos en la participación en el Proyecto *Resenha da Prevenção*. El nivel de influencia interpersonal fue predominante.

**Consideraciones Finales:**

desde la percepción de los adolescentes, el Proyecto *Resenha da Prevenção* promueve el desarrollo personal y social al ofrecer un espacio seguro de aprendizaje y reflexión.

## INTRODUCTION

The consumption of alcohol and other drugs among adolescents represents a global public health concern, especially due to its significant impacts on mental health, academic performance, social integration, and the potential for health risk behaviors^(1-3)^. The effects of early substance use during adolescence may persist into adulthood, being associated with functional brain alterations^(4)^, increased risk of mental health disorders^(5)^, and anxiety in adult life^(6)^.

The World Health Organization (WHO) reports that 23.5% of young people aged 15 to 19 consume alcohol globally, with higher prevalence in Europe (45.9%) and the Americas (43.9%)^(7)^. In Brazil, alcohol is the most commonly used psychoactive substance among adolescents aged 12 to 17 (34.3% have tried it at least once in their lifetime and 22.2% in the past 12 months), followed by regular cannabis use (12.5%) and cocaine (2.5%)^(8,9)^. Data from the National School Health Survey (2019) revealed that 3.3% of Brazilian adolescents engage in polysubstance use, a phenomenon influenced by sociodemographic, family, behavioral, and stress related factors, highlighting the need to implement prevention strategies during adolescence^(10)^.

Studies indicate that health promotion programs and projects based on sports activities not only foster physical and mental well being but also contribute to strengthening social skills and increasing the sense of belonging in vulnerable communities^(11,12)^. They also promote positive peer interactions, serving as a protective factor against substance use^(13,14)^.

The *Resenha da Produção* project, openly translated as “Conversations about alcohol and other drugs,” is a Brazilian initiative promoted by the Government of the State of Ceará that aims to prevent the use of alcohol and other drugs through an integrated approach that combines sports, education, and the strengthening of community bonds. Launched in 2022, the project trains community sports facilitators to act as prevention agents, leading discussion circles focused on the healthy development of children and adolescents^(15)^.

To understand the perceptions of adolescents participating in the *Resenha da Prevenção* project, this study adopts the Socio Ecological Model by McLeroy et al. (1988)^(16)^ as its theoretical framework. This model examines environmental influences on health conditions and human behaviors across different levels (intrapersonal, interpersonal, organizational, community, and policy). It enables the interpretation of how the social context shapes adolescents’ perceptions and behaviors and can contribute to the development of sustainable strategies for preventing health problems^(17-19)^.

In addition, the present study aligns with the United Nations Sustainable Development Goals (SDGs)^(20)^, particularly SDG 3 (Good Health and Well Being), target 3.5, which emphasizes the importance of preventing and treating substance abuse. By integrating sports and education as prevention tools, the study also reinforces the need for inclusive and accessible education, consistent with SDG 4 (Quality Education).

While the literature on substance use prevention among adolescents is extensive, the integration of adolescents’ own perspectives combined with a detailed socio ecological analysis of a specific program such as the *Resenha da Prevenção* Project is less common and represents an advancement^(21)^. The qualitative evaluation of the project may contribute to strengthening future evidence based interventions^(22)^. Therefore, this study formulated the following research question: How do adolescents participating in the *Resenha da Prevenção* Project perceive alcohol and other drug use and their participation in the project?

## OBJECTIVES

To analyze adolescents’ perceptions of their participation in the *Resenha da Prevenção* Project and their views on the consumption of alcohol and other drugs, from the perspective of the Socio Ecological Model.

## METHODS

### Ethical aspects

The study was conducted in accordance with national and international ethical guidelines and was approved by the Ethics Committee of the *Universidade Federal do Ceará* (UFC, Federal University of Ceará), whose approval statement is attached to this submission. The adolescents also signed the Free and Informed Assent Form. To ensure confidentiality and anonymity of the participants, an alphanumeric identification system was used.

### Type of study and theoretical framwork

This is a descriptive study with a qualitative approach, using the Consolidated Criteria for Reporting Qualitative Research (COREQ) checklist, validated for Brazil^(23)^. The Socio Ecological Model by McLeroy et al. (1988)^(16)^ was adopted as the theoretical framework, identifying five levels of influence that sustain, maintain, or affect human behaviors: intrapersonal, interpersonal, organizational, community, and policy levels (Figure 1).

**Figure 1:**
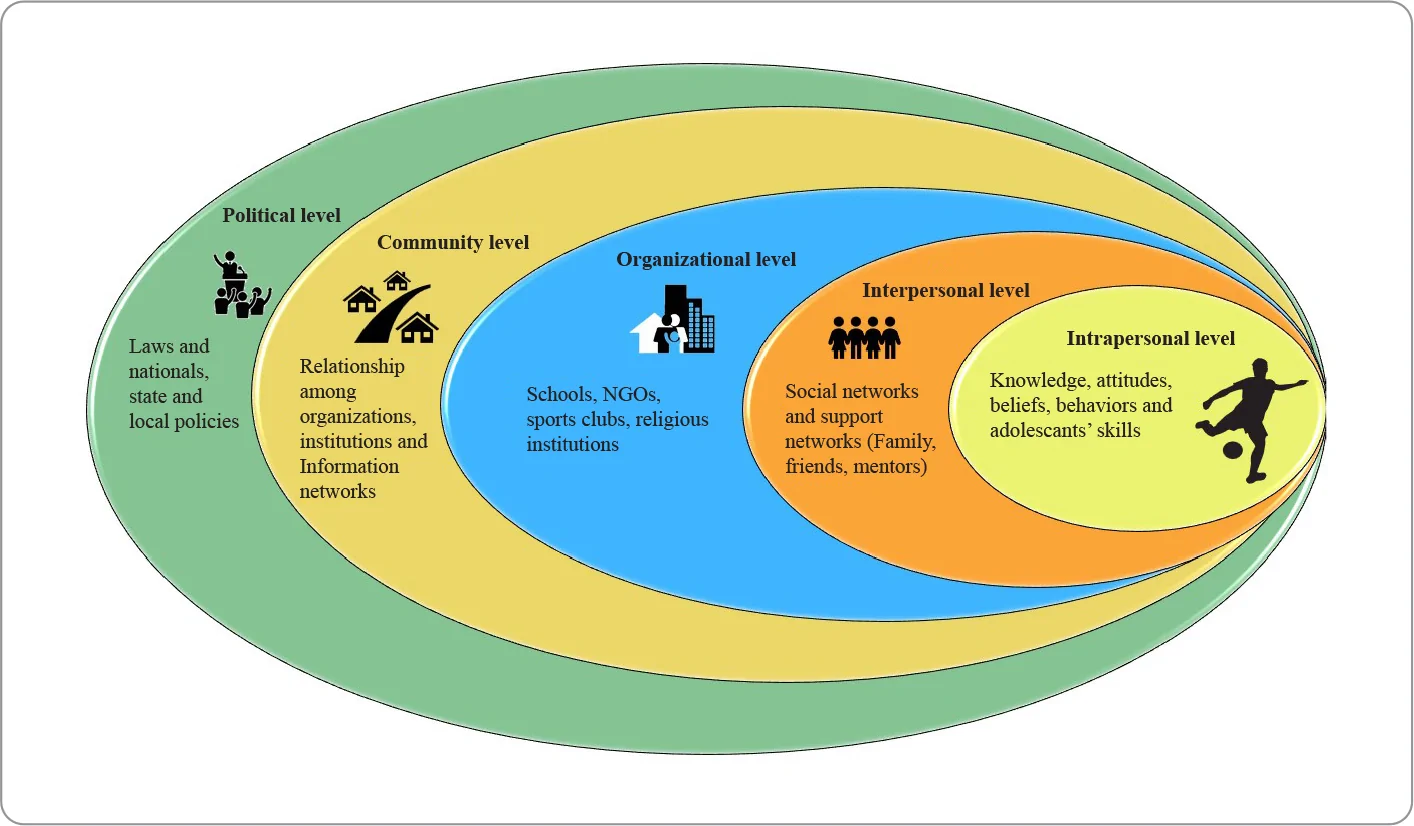
The Socio Ecological Model (adapted) (McLeroy, 1988)

### Study setting

The present study was developed based on the implementation of the *Resenha da Prevenção* Project. The study was carried out across four municipalities in the state of Ceará. Data collection took place in community settings that offered sports activities, such as courts and stadiums.

The methodology of the *Resenha da Prevenção* Project is grounded in an integrated and participatory approach that combines a sports activity freely chosen by the adolescent with educational discussion circles. Each discussion circle lasts at least 50 minutes and addresses topics relevant to the integral development of young people, such as childhood and adolescence, healthy habits, mental health, human rights, a culture of peace, education and communication, environmental preservation, education, education, technology, social media, life projects, and adolescent leadership.

Currently, the project covers 32 municipalities in Ceará with populations exceeding 50,000 inhabitants, all of which have Municipal Drug Policy Councils and, therefore, since its creation by the Executive Secretariat for Drug and Citizenship Policies of the Government of Ceará, receive integrated prevention actions. For the implementation of these actions, the project is carried out through a civil society organization called the Ceará Institute of Art and Citizenship (IAC CE). With a total population above 50,000 inhabitants, the project reaches approximately 4,000 adolescents through various sports activities, such as soccer, volleyball, judo, skateboarding, among others, across 14 planning regions of Ceará.

The activities are supported by a multidisciplinary team linked to the Health Care Network, composed of nurses, psychologists, physical educators, social workers, among others, with the aim of providing comprehensive support to participants. To ensure broad reach and strengthen community based actions, the project conducts a selection process for local leaders, known as Sports and Prevention Promotion Agents. These agents are responsible for conducting sports activities and discussion circles in their communities after receiving specific training, educational materials, and continuous technical support from the project’s coordination team.

### Participants of the study

The study participants were adolescents aged 10 to 19 who were enrolled in the *Resenha da Prevenção* Project in the selected municipalities and who regularly participated in the sports activities.

### Data organization and collection

Data were collected between May and June 2025 through eight focus groups, each lasting an average of 50 minutes. Each adolescent participated in only one focus group, composed of an average of 12 to 15 participants. The discussions, facilitated by an experienced researcher and a research assistant, followed a semi structured guide that explored adolescents’ perceptions of the project, as well as their views on alcohol and drug use, peer interactions, and relationships with family and the community.

Participants were also invited to suggest improvements to the activities, contributing to the project’s evaluation. The focus groups were conducted before the sports activities, in accessible and comfortable locations, following the principles of Barbour (2009)^(24)^. All sessions were audio recorded, fully transcribed, and anonymized to preserve participant confidentiality. Saturation was reached when information began to repeat, making the collection of new data unnecessary^(25)^.

### Data Analysis

The statements from the focus groups were transcribed by the research team, followed by double checking to ensure completeness and accuracy of the accounts. Data analysis was conducted manually and validated by four coders. Reflexive thematic analysis was employed according to the approach proposed by Braun and Clarke (2006)^(26)^, following the steps: (1) familiarization with the data; (2) generation of initial codes; (3) search for themes; (4) review of themes; (5) definition and naming of themes; and (6) production of the report.

After data analysis, themes and subthemes emerged and were organized based on the five levels of influence of McLeroy’s (1988)^(16)^ Socio Ecological Model, the theoretical framework adopted for interpreting the results. To systematize the responses, a thematic map (Figure 2) was developed, representing the themes identified through the analysis and highlighting the interrelationships among the different levels of influence present in the adolescents’ perceptions. This categorization was guided by the questions included in the focus group script, allowing the responses to be organized according to the identified levels of influence.

**Figure 2:**
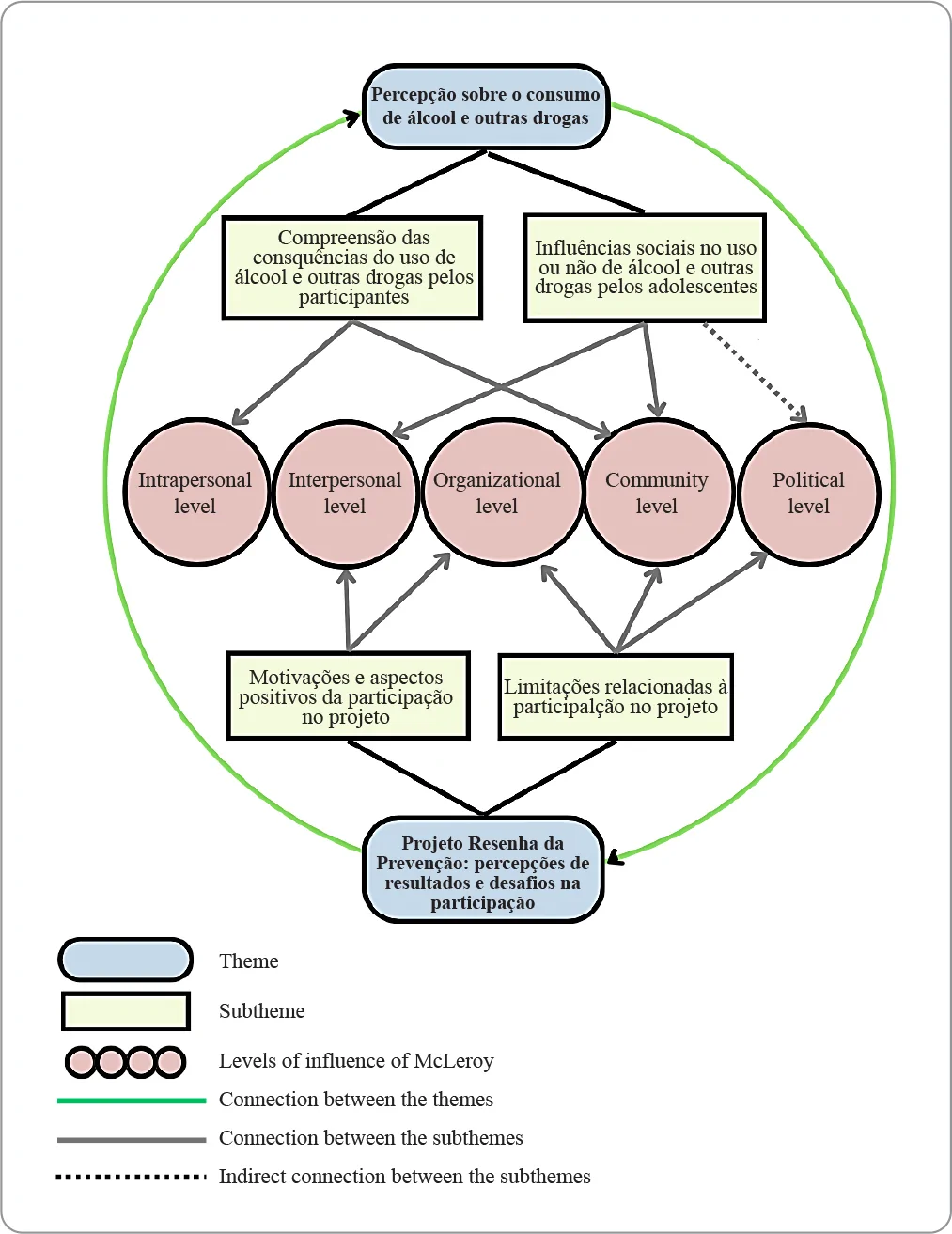
Thematic map representing the themes and subthemes of the analysis, Fortaleza, Ceará, Brazil, 2025

## RESULTS

A total of 97 adolescents participated in the focus groups, of whom 66 (68.04%) were male and 31 (31.96%) were female. The predominant age was 14 years (48.00%). All participants were students, with 76 (78.35%) enrolled in elementary school and 21 (21.65%) in high school. Regarding sports practices, the most frequently mentioned modalities were soccer (32; 32.99%), volleyball (26; 26.80%), *futsal* (25; 25.77%), and skateboarding (14; 14.43%). The focus groups were organized and identified using the codes: FG1, FG2, FG3, FG4, and FG5.

The inductive analysis of the adolescents’ statements revealed two central themes, organized according to subthemes, codes, and the levels of influence of McLeroy’s (1988)^(16)^ Socio Ecological Model. The first theme, “Perceptions of alcohol and other drug use”, includes the subthemes “Understanding the risks of alcohol and other drug use among adolescents” and “Social influences on adolescents’ use or non use of alcohol and other drugs”.

The second theme, “Perceptions of outcomes and challenges in participating in the *Resenha da Prevenção* Project”, is subdivided into “Motivations and positive aspects of participating in the project” and “Limitations related to participation in the project”. These findings are systematized in Chart 1, detailing the emerging codes and the levels of influence associated with each category of analysis.

**Chart 1:** Socio ecological levels in adolescents’ perceptions of alcohol and other drug use and participation in the *Resenha da Prevenção* project, Fortaleza, Ceará, Brazil, 2025

Theme	Subtheme	Coding	Level of influence
Perceptions of alcohol and other drug use	Understanding the risks of alcohol and other drug use among adolescents	Impacts of drug use on mental and physical health	Intrapersonal - Comunitary
		Future consequences of use	
		Importance of awareness	
	Social influences on adolescents’ use or non-use of alcohol and other drugs	Direct exposure to substances	Interpersonal - Comunitary
		Influence of peers and the social environment	
		Alternatives to substance use through sports	
Perceptions of outcomes and challenges in participating in the *Resenha da Prevenção* Project	Motivations and positive aspects of participating in the project	Development of values and skills	Organizational - Interpersonal
		Discovery of talents	
		Role of the project in socialization and well-being	
	Limitations related to participation in the project	Inclusion challenges	Organizational - Comunitary
		Need for more resources and infrastructure	
		Adjustments to the dynamics of activities	
		Perception of governmental and community support for the project	Political

### Perceptions of alcohol and other drug use

The consumption of alcohol and other drugs among adolescents is a complex phenomenon influenced by individual, social, and environmental factors. The participants demonstrated an understanding of the risks involved, also recognizing the social influences that may encourage or inhibit such behavior. At the intrapersonal level, individual perceptions of risk were most prominent, while at the interpersonal and community levels, the influence of support networks and the social environment stood out.

In the subtheme Understanding the risks of alcohol and other drug use among adolescents, they expressed concern about the negative effects on health, social life, and future professional prospects, associating substance use-especially marijuana-with mental health problems and organic complications. They also emphasized the importance of education and dialogue, particularly through discussion circles, as a strategy for awareness and prevention.

*Because on the day I’m here* […] *I could be doing other things, right? I could be doing wrong things. I could be doing the right things too, but I came here. To train, to watch.* (FG1)

*Teenagers who use marijuana face problems like depression, anxiety, and kidney problems. And black lungs from smoking cigarettes.* (FG1)

[…] *jail* […] *it will ruin your future!* [Alcohol and drugs]. (FG4)

*This is wrong because the child is underage. Even if they were older, they shouldn’t be using it! There are many people who died young, like at 30 years old. That’s young to me. It’s not something that will happen now, but it will happen in the future.* (FG5)

In the subtheme Social influences on adolescents’ use or non use of alcohol and other drugs, the social context surrounding experimentation and drug consumption emerged as a recurring topic. The adolescents reported situations in which they were directly exposed to substances, often without a clear understanding of the risks involved.

*There’s a guy right over there who walks around with a jug of loló and comes up to us asking, ‘Do you want some?’. Then I saw people just asking for it, and at one point he left, went into an apartment, and came back with the jug even fuller. For them, it’s like water.* (FG1)

*Some friends lead us down that path.* (FG1)

*We live in a place that is surrounded by this* [drugs]. (FG3)

[…] *it was very interesting because teenagers nowadays hang out with a lot of the wrong people and end up getting lost in the wrong world, right?* (FG3)

*Give advice, don’t let them hang out with the wrong people, focus on school, be careful* [About the family’s role]. (FG4)

### Perceptions of outcomes and challenges in participating in the *Resenha da Prevenção* Project

The *Resenha da Prevenção* Project was perceived by adolescents as a positive initiative for preventing drug use, promoting personal development, and strengthening socialization. Their statements highlighted the institutional role in the activities and the importance of interpersonal and community levels, expressed through interactions and mutual support among participants.

In the subtheme Motivations and positive aspects of participating in the project, the adolescents emphasized learning related to prevention, respect, and responsibility, as well as valuing the project as a space to discuss social issues, discover talents and professional interests, and receive emotional and motivational support. These elements reinforce the role of interpersonal relationships in youth development.

*We learn new things* […] *to learn about prevention, care, and respect.* (FG1)

*I found it very necessary because we addressed several issues about respect for skin color, sexuality, and that is extremely necessary for us.* (FG2)

*I chose this project because I wanted to distract myself a bit from situations in my life, and I ended up discovering my passion - I want to become a professional player.* (FG2)

*It got me out of the house more, I met good people, and even though it’s a volleyball project, it teaches you things about life. And when I was sad or upset because I made mistakes or made my team lose, the coach didn’t yell at me. He said: keep your head up, take your mistakes and learn from them, because we learn more from mistakes than from successes - and that changed me a lot.* (FG2)

*It was interesting and very good to raise awareness in people about the mistakes they make and to avoid making them again* […] *I found it very necessary because we addressed several issues about respect for skin color, sexuality, and that is extremely necessary for us* […] *it helped me socialize* […] *it helped me with my sedentary lifestyle.* (FG2)

*Talking about the discussion circles, I found it very important that they talked about alcohol, education, coexistence, because there are people like the ones here who don’t know about this. When you’re already an adult, you understand the differences in growth, in social classes. Some may grow up thinking it’s normal, but that’s not normal* […]. (FG5)

*When a delicate situation happened in volleyball, we had a circle and discussed the topic - how we should behave - and from that moment on, everyone’s behavior improved, since we talked about respect and prejudice as well.* (FG2)

*At the end of practice, we participated in talks where they discussed bullying,* [racial] *prejudice, and prejudice against LGBT+ people, and we talked in a very open way. It was incredible because everyone had the chance to speak and listen, and the moment itself was amazing.* (FG2)

*The groups were great for having someone to vent to and talk to when needed*. (FG2)

The subtheme Limitations related to participation in the project highlights that, although the project brings important benefits, adolescents reported challenges such as issues related to inclusion and infrastructure, as well as the need for more dynamic training sessions and better access to equipment. The discussion circles were viewed as essential spaces for dialogue and awareness raising, although some adjustments were suggested to improve the activity. The positive impact of these discussions was consistently emphasized.

*Sometimes there’s a bit of exclusion, selectiveness, from people who already have a lot of ease with volleyball and from people who don’t have that much ease, but it’s okay, because we’re managing to improve with the training*. (FG2)

*What needs to improve are the equipment and investments - shoes and materials - so we can have better conditions to play well.* (FG2)

[…] *too time consuming, the discussion circles should be more objective.* (FG3)

*Something I didn’t like very much was the excess of repetitive training; I wanted it to be more dynamic.* (FG2)

*There should be a water fountain here too* […] *the space is small* […] *add more topics in the discussion circles about everyday life.* (FG5)

## DISCUSSION

In light of the Socio Ecological Theory, the findings of this study can be understood across different levels of influence that collectively shape adolescent behavior. At the intrapersonal level, individual awareness and clarity regarding the consequences of substance use stand out. At the interpersonal level, social bonds, respect, and emotional support emerge as essential elements for strengthening support networks. At the community level, the role of collective initiatives, such as the project analyzed, becomes evident, as they promote spaces for dialogue and belonging. At the policy level, the importance of institutional and governmental contexts is highlighted, particularly those that ensure infrastructure, resources, and continuity of actions, reinforcing the relevance of public policies focused on adolescent health. For nursing, this perspective broadens the scope of practice by demonstrating that preventing alcohol and other drug use among adolescents requires integrated actions capable of articulating health education, the strengthening of interpersonal relationships, and community engagement.

The statements of adolescents participating in the *Resenha da Prevenção* Project revealed motivations such as learning, strengthening bonds, and socialization. Elements such as respect and coexistence fostered a sense of belonging and support, promoting socio emotional skills and support networks. International studies confirm that preventive programs grounded in community based and educational experiences reduce substance use among adolescents^(27)^.

At the intrapersonal level, associations emerged between substance use and harm to mental and physical health, as well as long term risks such as loss of future prospects. This recognition supports protective decision making when reinforced by consistent educational strategies^(28,29)^. Participation in the project functioned as a catalyst for awareness, strengthening autonomy. Interventions led by nurses have already demonstrated effectiveness in reducing alcohol consumption among adolescents^(30)^.

The interpersonal level was marked by the influence of peers and family members. Many adolescents reported exposure to drugs in social contexts, but they also identified support from friends and relatives, aligning with the literature that identifies peers as both a risk and a protective factor^(31,32)^. Open family communication reduces substance experimentation^(32,33)^.

At the organizational level, adolescents reported limitations related to infrastructure and scarce resources, but they also recognized the potential of educational and sports initiatives to promote self esteem and new perspectives. The effectiveness of such projects depends on adequate structure and encouragement of adolescent protagonism^(34)^. The challenges identified reinforce the importance of continuous adjustments in preventive strategies. Programs that combine educational content with sports practice enhance the effectiveness of interventions^(35)^. To ensure adherence, they require constant monitoring, regular evaluations, and flexibility^(35-37)^.

At the community level, the project was recognized as a space for socialization and dialogue, especially through discussion circles addressing drugs and mental health. Recreational and preventive spaces reduce substance use^(38)^. Accessible sports and cultural projects decrease vulnerability to alcohol and other drugs^(35)^. Experiences that combine sports and dialogue strengthen adolescent agency^(34,38)^. Although less frequently mentioned, the policy level appeared in perceptions regarding governmental support, reinforcing the need for sustainable public policies with adequate funding and integration between health and education sectors. Studies show that long term, culturally adapted programs are more effective^(36-37)^.

The findings show that adolescents’ perceptions involve relational, community, and structural dimensions, which distinguishes this study from others focused solely on sports practices by valuing adolescents’ voices^(39)^. The articulation with the Socio Ecological Model highlights the complexity of the influences on adolescent behavior and provides important insights for nursing practice. Transversally, the relevance of nursing stands out in the planning and implementation of educational and preventive interventions within both family and community contexts. Addressing alcohol and other drug use requires actions that consider mental health problems, substance misuse, and school performance, achieving greater effectiveness in promoting resilience and healthy development^(40)^. Strategies led by nurses, grounded in continuous educational approaches and the strengthening of support networks, have proven effective in reducing alcohol consumption and preventing risk behaviors^(30,41)^. In this sense, nursing assumes a strategic role by integrating educational practices with community engagement, establishing itself as a key field for expanding the reach and effectiveness of preventive actions among adolescents.

### Study limitations

A limitation of this study relates to the timing of the focus groups, which were conducted before the sports activities. Furthermore, because the sample was drawn from four municipalities in the state of Ceará, the findings cannot be generalized to other sociocultural contexts. The absence of a longitudinal approach also limits the understanding of the project’s long term effects on adolescents’ trajectories and suggests the need for ongoing evaluation of the initiative.

### Contributions to the area

The results contribute to the development of more effective and culturally sensitive interventions, valuing practices such as sports and discussion circles in preventing substance use. Such strategies can be incorporated into primary care and nursing actions in vulnerable contexts. In addition, identifying the project’s challenges provides insights for improving similar programs and reinforces the strategic role of nurses in promoting adolescent health.

## FINAL CONSIDERATIONS

The analysis, grounded in McLeroy’s socio ecological model, showed that adolescents’ perceptions of alcohol and other drug use are broadly shaped by individual, interpersonal, organizational, community, and policy level factors. The actions developed by the *Resenha da Prevenção* Project demonstrated a central role in promoting the physical, social, and emotional well being of participants. However, the continuity and sustainability of the initiative require overcoming logistical challenges, as well as conducting ongoing evaluations anchored in the adolescents’ own perceptions, in order to ensure the effectiveness and long term impact of the intervention.

## Data Availability

The research data are available only upon request.
