# Supplementary material for: Project Resenha da Prevenção and drug use: adolescents’ perceptions from a socio ecological perspective
Source: Rev Bras Enferm. 2026 Nov 6;79:e20250342. doi: 10.1590/0034-7167-2025-0342 (PMC13743046; doi:10.1590/0034-7167-2025-0342)
Supplement: Translation

# Translation: Projeto Resenha da Prevenção e consumo de drogas: percepções de adolescentes sob a ótica socioecológica

**DOI:** 10.1590/0034-7167-2025-0342pt

**Authors:** Brenda Pinheiro Evangelista, Thais Rodrigues de Albuquerque, Kirley Kethellen Batista Mesquita, Mariana Sales Bastos, Priscila de Souza Aquino, Lorena Pinheiro Barbosa, Patrícia Neyva da Costa Pinheiro, Fabiane do Amaral Gubert

**Affiliations:** I Universidade Federal do Ceará. Fortaleza, Ceará, Brasil

**Keywords:** Promoção da Saúde, Adolescente, Criança, Instalações Esportivas e Recreacionais, Prevenção Primária.

## Abstract

**Objetivos:**

analisar as percepções de adolescentes sobre a participação no Projeto Resenha da Prevenção, e acerca do consumo de álcool e outras drogas, sob a ótica do Modelo Socioecológico.

**Métodos:**

estudo qualitativo realizado junto a 97 adolescentes de 10 a 19 anos, cadastrados no projeto Resenha da Prevenção em quatro municípios do estado do Ceará, Brasil, entre maio e junho de 2025. Os dados, coletados por meio da técnica de grupo focal, foram submetidos à análise temática reflexiva e analisados a partir do Modelo Socioecológico.

**Resultados:**

emergiram dois temas centrais: Percepção sobre o consumo de álcool e outras drogas e Percepções de resultados e desafios na participação do Projeto Resenha da Prevenção. O nível de influência interpessoal foi predominante.

**Considerações Finais:**

na percepção dos adolescentes, o projeto Resenha da Prevenção promove o desenvolvimento pessoal e social ao proporcionar um espaço seguro de aprendizado e reflexão.

## INTRODUÇÃO

O consumo de álcool e outras drogas entre adolescentes representa uma preocupação global de saúde pública, especialmente devido aos impactos significativos na saúde mental, desempenho acadêmico, inserção social e potencial para comportamentos de risco à saúde^(1-3)^. Os efeitos do consumo precoce dessas substâncias durante a adolescência podem repercutir até a vida adulta, sendo associados a alterações funcionais do cérebro^(4)^, aumento do risco de transtorno de saúde mental^(5)^ e ansiedade na vida adulta^(6)^.

A Organização Mundial da Saúde (OMS) indica que 23,5% dos jovens entre 15 e 19 anos consomem álcool globalmente, com maior prevalência na Europa (45,9%) e Américas (43,9%)^(7)^. No Brasil, o álcool é a substância psicoativa mais consumida entre adolescentes de 12 a 17 anos (34,3% experimentaram ao longo da vida e 22,2% nos últimos 12 meses), seguido pelo uso regular de cannabis (12,5%) e cocaína (2,5%)^(8,9)^. Dados da Pesquisa Nacional de Saúde do Escolar (2019) revelaram que 3,3% dos adolescentes brasileiros apresentam policonsumo, um fenômeno influenciado por fatores sociodemográficos, familiares, comportamentais e eventos estressores, demonstrando a necessidade de se implementar estratégias de prevenção na adolescência^(10)^.

Estudos indicam que programas e projetos de promoção da saúde baseados em práticas esportivas não apenas promovem bem-estar físico e mental, mas contribuem para o fortalecimento de habilidades sociais e aumento do senso de pertencimento em comunidades vulneráveis^(11,12)^ e também promovem interações sociais positivas entre pares, constituindo-se como fator protetor ao uso de substâncias^(13,14)^.

O Projeto Resenha da Prevenção é uma iniciativa brasileira promovida pelo Governo do Estado do Ceará que visa prevenir o uso de álcool e outras drogas por meio de uma abordagem integrada que alia esporte, educação e fortalecimento de vínculos comunitários. Lançado em 2022, o projeto capacita facilitadores de práticas esportivas das comunidades para atuar como agentes de prevenção, conduzindo rodas de conversas focadas no desenvolvimento saudável de crianças e adolescentes^(15)^.

Para compreender as percepções dos adolescentes participantes do projeto Resenha da Prevenção, este estudo adota como referencial teórico o Modelo Socioecológico de McLeroy et al. (1988)^(16)^, que analisa influências do ambiente em condições de saúde e comportamentos humanos em diferentes níveis (intrapessoal, interpessoal, organizacional, comunitário e político). Esse modelo permite interpretar como o contexto social molda as percepções e os comportamentos dos adolescentes, e pode contribuir para a formulação de soluções sustentáveis de prevenção de agravos à saúde^(17-19)^.

Além disso, o presente estudo alinha-se aos Objetivos de Desenvolvimento Sustentável (ODS) da Organização das Nações Unidas^(20)^, destacando-se o ODS 3 (Saúde e Bem-Estar) na meta 3.5, que enfatiza a importância da prevenção e do tratamento do abuso de substâncias. Ao integrar esporte e educação como ferramentas de prevenção, reforça-se a necessidade de uma educação inclusiva e acessível, em consonância com o ODS 4 (Educação de Qualidade).

Enquanto a literatura sobre prevenção do uso de substâncias em adolescentes é vasta, a integração da perspectiva dos próprios jovens, aliada a uma análise socioecológica detalhada de um programa específico como o Projeto Resenha da Prevenção, é menos comum e representa um avanço^(21)^. A avaliação qualitativa do projeto pode contribuir para o fortalecimento de futuras intervenções baseadas em evidência^(22)^. Dessa forma, neste estudo delineou-se a seguinte questão: Como adolescentes participantes do Projeto Resenha da Prevenção percebem o consumo de álcool e outras drogas e sua participação no projeto?

## OBJETIVOS

Analisar as percepções de adolescentes sobre a participação no Projeto Resenha da Prevenção, e acerca do consumo de álcool e outras drogas, sob a ótica do Modelo Socioecológico.

## MÉTODOS

### Aspectos éticos

O estudo foi conduzido conforme diretrizes éticas nacionais e internacionais e aprovado pelo Comitê de Ética da Universidade Federal do Ceará (UFC), cujo parecer está anexado à presente submissão. Os adolescentes ainda assinaram o Assentimento Livre e Esclarecido (TALE). Para garantia do sigilo e anonimato dos participantes, foi empregada uma identificação alfanumérica.

### Tipo de estudo e referencial teórico

Trata-se de um estudo descritivo, com abordagem qualitativa, utilizando a lista de verificação *Consolidated Criteria for Reporting Qualitative Research* (COREQ) validada para o Brasil^(23)^. Adotou-se como referencial teórico o Modelo Socioecológico de McLeroy et al. (1988)^(16)^, o qual identifica cinco níveis de influência que sustentam, mantém ou afetam os comportamentos humanos: nível intrapessoal, interpessoal, organizacional, comunitário e político (Figura 1).

**Figura 1:** Modelo Socioecológico (adaptado) (McLeroy, 1988)

### Cenário do estudo

O presente estudo foi desenvolvido a partir da implementação do Projeto Resenha da Prevenção. O estudo foi realizado abrangendo quatro municípios do Ceará. A coleta de dados ocorreu em locais comunitários que ofereciam atividades esportivas, como quadras e estádios.

A metodologia do Projeto Resenha da Prevenção baseia-se em uma abordagem integrada e participativa, que combina a realização de uma atividade esportiva de livre escolha do adolescente com rodas de conversa educativas. Cada roda de conversa possui duração mínima de 50 minutos e aborda temáticas relevantes ao desenvolvimento integral dos jovens, como infância e adolescência, hábitos saudáveis, saúde mental, direitos humanos, cultura de paz, educomunicação, preservação ambiental, educação, tecnologia, redes sociais, projeto de vida e protagonismo do adolescente.

Atualmente, o projeto abrange 32 municípios do Ceará, com população total superior a 50.000 habitantes, que contam com Conselhos Municipais de Políticas sobre Drogas e, assim, desde sua criação pela Secretaria Executiva de Políticas sobre Drogas e Cidadania do Governo do Ceará, recebem ações integradas de prevenção. Para a implementação das ações, estas são realizadas mediante uma organização da sociedade civil intitulada Instituto de Arte e Cidadania do Ceará (IAC-CE). Com uma população total superior a 50.000 habitantes, o projeto atinge cerca de 4.000 adolescentes por meio de diversas atividades esportivas, como futebol, vôlei, *judô, skate*, entre outras, em 14 regiões de planejamento do Ceará.

As atividades são apoiadas por uma equipe multiprofissional vinculada à Rede de Atenção à Saúde, composta por enfermeiros, psicólogos, educadores físicos, assistentes sociais, entre outros, com o intuito de oferecer suporte integral aos participantes. Para garantir capilaridade e fortalecimento das ações comunitárias, o projeto realiza processo seletivo de líderes locais, denominados Agentes Promotores de Esporte e Prevenção. Esses agentes são responsáveis por conduzir as atividades esportivas e rodas de conversa em suas comunidades, após receberem formação específica, materiais didáticos e suporte técnico contínuo da equipe de coordenação do projeto.

### Participantes do estudo

Os participantes do estudo foram adolescentes, entre 10 e 19 anos, cadastrados no Projeto Resenha da Prevenção nos municípios selecionados, e que participavam regularmente das atividades esportivas.

### Coleta e organização dos dados

Os dados foram coletados entre maio e junho de 2025 por meio de oito grupos focais, cada um com duração média de 50 minutos. Cada adolescente participou de apenas um grupo focal, composto por uma média de 12 a 15 participantes. As discussões, mediadas por pesquisador experiente e assistente de pesquisa, seguiram um roteiro semiestruturado que abordava a percepção dos adolescentes sobre o projeto, bem como suas concepções acerca do consumo de álcool e drogas, das interações entre pares e das relações com a família e comunidade.

Os participantes foram convidados a sugerir melhorias para as atividades, contribuindo para a avaliação do projeto. Os grupos focais ocorreram antes das atividades esportivas, em locais acessíveis e confortáveis, seguindo os princípios de Barbour (2009)^(24)^. As sessões foram gravadas em áudio, transcritas integralmente e anonimizadas para preservar a confidencialidade dos participantes. A saturação foi alcançada quando as informações começaram a se repetir, tornando desnecessária a aquisição de novas informações^(25)^.

### Análise dos dados

As falas provenientes dos grupos focais foram transcritos pela equipe de pesquisa, e posteriormente realizou-se dupla checagem para garantir a completude e precisão dos relatos. A análise dos dados foi realizada de forma manual e contou com a validação de quatro codificadores. Utilizou-se a análise temática reflexiva conforme a abordagem de Braun e Clarke (2006)^(26)^, procedendo às etapas: 1) familiarização com os dados; 2) geração de códigos iniciais; 3) busca por temas; 4) revisão dos temas; 5) definição e nomeação dos temas; e 6) produção do relatório.

Após a análise dos dados, emergiram temas e subtemas organizados com base nos cinco níveis de influência do Modelo Socioecológico de McLeroy (1988)^(16)^, referencial teórico adotado para a interpretação dos resultados. Para sistematizar as respostas, foi elaborado um mapa temático (Figura 2), representando os temas identificados a partir da análise e evidenciando a inter-relação entre os diferentes níveis de influência presentes nas percepções dos adolescentes. Essa categorização foi orientada pelas questões do roteiro utilizado nos grupos focais, permitindo organizar as respostas conforme os níveis de influência identificados.

**Figura 2:** Mapa temático de representação dos temas e subtemas da análise, Fortaleza, Ceará Brasil, 2025

## RESULTADOS

Dos grupos focais realizados participaram um total de 97 adolescentes, sendo 66 (68,04%) do gênero masculino e 31 (31,96%) do gênero feminino. A idade predominante foi de 14 anos (48,00%). Todos os participantes eram estudantes, com 76 (78,35%) matriculados no ensino fundamental e 21 (21,65%) no ensino médio. No que se refere às práticas esportivas, destacaram-se o futebol, 32 (32,99%), o vôlei, 26 (26,80%), o futsal, 25 (25,77%) e o *skate,* 14 (14,43%), como as modalidades mais mencionadas. Os grupos focais foram organizados e identificados por meio dos códigos: GF1, GF2, GF3, GF4 e GF5.

A análise indutiva realizada a partir das falas dos adolescentes revelou dois temas centrais, organizados conforme os subtemas, códigos e níveis de influência do Modelo Socioecológico de McLeroy (1988)^(16)^. O primeiro tema, “Percepção sobre o consumo de álcool e outras drogas”, contempla os subtemas “Compreensão dos riscos do uso de álcool e outras drogas pelos adolescentes” e “Influências sociais no uso ou não de álcool e outras drogas pelos adolescentes”.

O segundo tema, “Percepções de resultados e desafios na participação do Projeto Resenha da Prevenção”, está subdividido em “Motivações e aspectos positivos da participação no projeto” e “Limitações relacionadas à participação no projeto “. Esses achados estão sistematizados no Quadro 1, detalhando os códigos emergentes e os níveis de influência associados a cada categoria de análise.

**Quadro 1:** Níveis socioecológicos nas percepções dos adolescentes sobre o consumo de álcool e outras drogas e a participação na Resenha da Prevenção, Fortaleza, Ceará, Brasil, 2025

Tema	Subtema	Código	Nível de Influência
Percepção sobre o consumo de álcool e outras drogas	Compreensão dos riscos do uso de álcool e outras drogas pelos adolescentes	Impactos do uso de drogas na saúde mental e física	Intrapessoal - Comunitário
		Consequências do uso no futuro	
		Importância da conscientização	
	Influências sociais no uso ou não de álcool e outras drogas pelos adolescentes	Exposição direta às substâncias	Interpessoal - Comunitário
		Influência dos pares e do ambiente social	
		Alternativas ao uso por meio do esporte	
Percepções de resultados e desafios na participação do Projeto Resenha da Prevenção	Motivações e aspectos positivos da participação no projeto	Desenvolvimento de valores e habilidades	Organizacional - Interpessoal
		Descoberta de talentos	
		Papel do projeto na socialização e no bem-estar	
	Limitações relacionadas à participação no projeto	Dificuldades de inclusão	Organizacional - Comunitário
		Necessidade de mais recursos e infraestrutura	
		Ajustes na dinâmica das atividades	
		Percepção sobre o apoio governamental e comunitário ao projeto	Político

### Percepção sobre o consumo de álcool e outras drogas

O consumo de álcool e outras drogas entre adolescentes é um fenômeno complexo, influenciado por fatores individuais, sociais e ambientais. Os participantes demonstraram compreender os riscos envolvidos, reconhecendo também as influências sociais que podem estimular ou inibir esse comportamento. No nível intrapessoal, destacaram-se as percepções individuais sobre os riscos, enquanto nos níveis interpessoal e comunitário sobressaiu a influência das redes de apoio e do meio social.

No subtema *Compreensão dos riscos do uso de álcool e outras drogas pelos participantes*, os adolescentes relataram preocupação com os efeitos negativos à saúde, à vida social e ao futuro profissional, associando o consumo, especialmente da maconha, a problemas de saúde mental e complicações orgânicas. Reforçaram, ainda, a importância da educação e do diálogo, especialmente por meio de rodas de conversa, como estratégia de conscientização e prevenção.

*É porque no dia de estar aqui* [...] *eu podia estar fazendo outras coisas, né? Podia estar fazendo coisas erradas. Podia fazer coisas certas também, mas eu vim para cá. Para treinar, para assistir*. (GF1)

*Os adolescentes que usam maconha enfrentam problemas como depressão, ansiedade e problemas nos rins. E pulmão preto por fumar cigarro*. (GF1)

[...] *cadeia* [...] *vai acabar com o futuro!* [Álcool e drogas]. (GF4)

*Isso é errado porque a criança é menor de idade. Mesmo se fosse de maior não era pra tá usando não! Tem muita gente que morreu cedo, tipo 30 anos. É novo pra mim. Não é uma coisa que vai acontecer agora, mas vai acontecer no futuro*. (GF5)

Já no subtema *Influências sociais no uso ou não de álcool e outras drogas pelos adolescentes* verifica-se que o contexto social na experimentação e no consumo de drogas foi temática recorrente. Os adolescentes relataram situações em que foram diretamente expostos a substâncias, muitas vezes sem que houvesse um entendimento claro sobre os riscos envolvidos.

*Tem um cara bem ali que anda com um galão de loló e chega na gente perguntando “Tu quer?”. Aí eu vi a galera só pedindo e teve uma hora que ele saiu, entrou num apartamento e depois voltou com o galão mais cheio ainda. Pra eles, é como água*. (GF1)

*Tem uns amigos que levam a gente pra esse caminho*. (GF1)

*A gente mora em um lugar que é rodeado disso* [drogas]. (GF3)

[...] *foi muito interessante porque os adolescentes hoje em dia andam com muitas pessoas erradas e acabam se perdendo no mundo errado, né?* (GF3)

*Dar conselho, não deixar andar com as pessoas erradas, focar nos estudos, cuidado* [Sobre o papel da família]. (GF4)

### Percepções de resultados e desafios na participação do Projeto Resenha da Prevenção

O Projeto Resenha da Prevenção foi percebido pelos adolescentes como uma iniciativa positiva para prevenir o uso de drogas, promover o desenvolvimento pessoal e fortalecer a socialização. As falas evidenciaram a atuação institucional nas atividades e a importância dos níveis interpessoal e comunitário, expressos nas interações e no apoio mútuo entre os participantes.

No subtema *Motivações e aspectos positivos da participação no projeto*, destacaram-se aprendizados relacionados à prevenção, respeito e responsabilidade, além da valorização do projeto como espaço para discutir questões sociais, descobrir talentos e interesses profissionais, e receber suporte emocional e motivacional, o que reforça o papel das relações interpessoais na formação dos jovens.

*A gente aprende coisas novas* [...] *para aprender sobre a prevenção, cuidado e respeito*. (GF1).

*Achei muito necessário, porque abordamos várias questões sobre respeito à cor da pele, à sexualidade e isso é extremamente necessário pra gente*. (GF2)

*Escolhi esse projeto porque queria me distrair um pouco das situações da minha vida e acabei descobrindo a minha paixão, quero ser jogador profissional.* (GF2)

*Me tirou mais de casa, conheci pessoas boas, e por mais que seja um projeto de vôlei, ele te ensina coisas sobre a vida, e quando eu estava triste ou abalada porque errava, ou fazia meu time perder, o treinador não brigava comigo, ele dizia: levanta a cabeça, pega teus erros e aprende com eles, porque com os erros aprendemos mais do que com os acertos e isso me fez mudar muito*. (GF2)

*Foi interessante e muito bom para conscientizar as pessoas, pelos erros que ela comete e para não cometer de novo* [...] *achei muito necessário, porque abordamos várias questões sobre respeito à cor da pele, à sexualidade e isso é extremamente necessário pra gente* [...] *me ajudou a socializar* [...] *me ajudou no sedentarismo*. (GF2)

*Falando sobre as rodas de conversa, achei bem importante tratarem sobre álcool, sobre educação, sobre convivência, pois tem pessoas como essas aqui que não sabem sobre isso. Você quando já é adulto, você sabe a diferença do crescimento, das classes. Algumas já podem crescer achando normal, mas isso não é o normal* [...]. *(GF5)*

*Quando aconteceu uma situação delicada no vôlei, fizemos uma roda e abordamos o tema, sobre como deveríamos nos comportar, e a partir desse momento, o comportamento de todos melhorou, já que abordamos o respeito e o preconceito também*. (GF2)

*Quando era fim de treino, a gente participava de palestras onde eles falavam sobre bullying, preconceito* [racial] *e preconceito com LGBT+, e falamos de maneira muito fluida e foi incrível porque todos tiveram a chance de falar e se escutar, e o momento em si foi muito incrível*. (GF2)

*As rodas foram ótimas para ter alguém pra desabafar e conversar quando precisa*. (GF2)

O subtema *Limitações relacionadas à participação no projeto* destaca que embora o projeto traga benefícios, os adolescentes relataram desafios como questões de inclusão e estrutura, além da necessidade de treinos mais dinâmicos e melhor acesso à equipamentos. As rodas de conversa foram vistas como fundamentais para o diálogo e conscientização, embora ajustes tenham sido sugeridos para melhorar a atividade. O impacto positivo dessas discussões foi destacado.

*Às vezes acontece um pouco de exclusão, seletividade, de pessoas que já tem muita facilidade com o vôlei e pessoas que não possuem tanta facilidade assim, mas tudo bem, porque estamos conseguindo evoluir com os treinos”.* (GF2)

*O que precisa melhorar são os equipamentos e investimentos, sapatos e materiais, para gente ter mais condições de jogar bem.* (GF2)

[...] *muito demorado, as rodas deveriam ser mais objetivas.* (GF3)

*Algo que eu não gostei tanto foi o excesso de treinos repetitivos, queria que fosse mais dinâmico.* (GF2)

*Era pra ter um bebedouro aqui também* [...] *o espaço é pequeno* [...] *colocar mais assuntos nas rodas sobre o cotidiano.* (GF5)

## DISCUSSÃO

À luz da Teoria Socioecológica, os achados deste estudo podem ser compreendidos a partir de diferentes níveis de influência que, em conjunto, moldam o comportamento dos adolescentes. No nível intrapessoal, destacam-se a conscientização individual e a clareza sobre as consequências do uso de substâncias. No nível interpessoal, sobressaem os vínculos sociais, o respeito e o suporte emocional, essenciais ao fortalecimento das redes de apoio. No nível comunitário, evidencia-se o papel de iniciativas coletivas, como o projeto analisado, que favorecem espaços de diálogo e pertencimento. Já no nível político, ressalta-se a importância de contextos institucionais e governamentais que assegurem infraestrutura, recursos e continuidade das ações, destacando a relevância de políticas públicas voltadas à saúde do adolescente. Para a enfermagem, essa perspectiva amplia o campo de atuação ao evidenciar que a prevenção ao uso de álcool e outras drogas entre adolescentes demanda ações integradas, capazes de articular a educação em saúde, o fortalecimento das relações interpessoais e o engajamento comunitário.

As falas dos adolescentes do Projeto Resenha da Prevenção revelaram motivações como aprendizado, fortalecimento de vínculos e socialização. Elementos como respeito e convivência favoreceram o acolhimento e o pertencimento, promovendo habilidades socioemocionais e redes de apoio. Estudo internacional confirma que programas preventivos baseados em vivências comunitárias e educativas reduzem o uso de substâncias entre adolescentes^(27)^.

No nível intrapessoal, emergiram associações entre consumo de substâncias e prejuízos à saúde mental e física, além de riscos de longo prazo, como perda de perspectivas de futuro. Esse reconhecimento favorece escolhas protetoras quando apoiado por estratégias educativas consistentes^(28,29)^. A participação no projeto funcionou como catalisador de conscientização, fortalecendo a autonomia. Intervenções conduzidas por enfermeiros já mostraram eficácia na redução do consumo de álcool entre adolescentes^(30)^.

O nível interpessoal foi marcado pela influência de pares e familiares. Muitos relataram exposição às drogas em contextos sociais, mas também identificaram apoio de amigos e familiares, confirmando a literatura que aponta os pares como fator de risco ou proteção^(31,32)^. A comunicação familiar aberta reduz a experimentação de substâncias^(32,33)^.

Na dimensão organizacional, os adolescentes relataram limitações de infraestrutura e escassez de recursos, mas também reconheceram o potencial de iniciativas educativas e esportivas em promover autoestima e novas perspectivas. A efetividade desses projetos depende de estrutura adequada e incentivo ao protagonismo do adolescente^(34)^. Os desafios apontados reforçam a importância de ajustes contínuos nas estratégias preventivas. Programas que unem conteúdo educativo e prática esportiva ampliam a eficácia das intervenções^(35)^. Para garantir adesão, exigem monitoramento constante, avaliações regulares e flexibilidade^(35-37)^.

No nível comunitário, o projeto foi reconhecido como espaço de socialização e diálogo, especialmente nas rodas de conversa sobre drogas e saúde mental. Espaços recreativos e preventivos reduzem o uso de substâncias^(38)^. Projetos esportivos e culturais acessíveis diminuem a vulnerabilidade ao álcool e outras drogas^(35)^. Experiências que combinam esporte e diálogo ampliam o protagonismo do adolescente^(34,38)^. Embora menos frequente, o nível político apareceu nas percepções sobre o apoio governamental, reforçando a necessidade de políticas públicas sustentáveis, com financiamento e integração entre saúde e educação. Estudos demonstram que programas duradouros e culturalmente adaptados são mais efetivos^(36,37)^.

Os achados mostram que as percepções dos adolescentes envolvem dimensões relacionais, comunitárias e estruturais, o que diferencia este estudo de outros focados apenas em práticas esportivas, ao valorizar a voz dos adolescentes^(39)^. A articulação com o Modelo Socioecológico evidencia a complexidade das influências sobre o comportamento do adolescente e fornece subsídios à prática da enfermagem. De forma transversal, destaca-se a relevância da enfermagem no planejamento e execução de intervenções educativas e preventivas nos contextos familiar e coletivo. O enfrentamento do uso de álcool e outras drogas requer ações que considerem problemas de saúde mental, abuso de substâncias e desempenho escolar, alcançando maior eficácia na promoção da resiliência e do desenvolvimento saudável^(40)^. Estratégias lideradas por enfermeiros, fundamentadas em abordagens educativas contínuas e fortalecimento das redes de apoio, mostram-se eficazes na redução do consumo de álcool e prevenção de comportamentos de risco^(30,41)^. Nesse sentido, a enfermagem assume papel estratégico ao integrar práticas educativas e articulação comunitária, consolidando-se como área fundamental para ampliar o alcance e a efetividade das ações preventivas junto aos adolescentes.

### Limitações do estudo

Uma limitação deste estudo refere-se ao momento da realização dos grupos focais, conduzidos antes das atividades esportivas. Ademais, por se tratar de uma amostra localizada em quatro municípios do Ceará, os achados não podem ser generalizados para outras realidades socioculturais. A ausência de uma abordagem longitudinal também limita a compreensão dos efeitos duradouros do projeto na trajetória dos adolescentes e sugere a necessidade de uma avaliação contínua do projeto.

### Contribuições para a área

Os resultados contribuem para o desenvolvimento de intervenções mais eficazes e culturalmente sensíveis, valorizando práticas como esportes e rodas de conversa na prevenção do uso de substâncias. Tais estratégias podem ser incorporadas à atenção primária e às ações de enfermagem em contextos vulneráveis. Além disso, a identificação dos desafios do projeto fornece subsídios para aprimorar programas semelhantes e reforça o papel estratégico do enfermeiro na promoção da saúde dos adolescentes.

## CONSIDERAÇÕES FINAIS

A análise, fundamentada no modelo socioecológico de McLeroy, evidenciou que a percepção dos adolescentes acerca do consumo de álcool e outras drogas é amplamente moldada por fatores de ordem individual, interpessoal, organizacional, comunitária e política. As ações desenvolvidas pelo Projeto Resenha da Prevenção demonstraram um papel central na promoção do bem-estar físico, social e emocional dos participantes. No entanto, a continuidade e sustentabilidade da iniciativa exigem a superação de desafios logísticos, bem como a realização de avaliações contínuas ancoradas nas percepções dos próprios adolescentes, de modo a assegurar a efetividade e a durabilidade dos impactos da intervenção.

## Data Availability

Os dados de pesquisa só estão disponíveis mediante solicitação.
